# Case Report of a Rare Nonpuerperal Uterine Inversion Managed with Uterosacral Ligament Hysteropexy

**DOI:** 10.1155/2021/4054924

**Published:** 2021-09-16

**Authors:** Ali Azadi, Alexandra Wolfe, Greg J. Marchand

**Affiliations:** ^1^University of Arizona, College of Medicine, 475 N 5th St, Phoenix, AZ 85004, USA; ^2^Star Urogynecology, 14155 N 83rd Ave, Suite 138, Peoria, AZ 85381, USA; ^3^OMS-IV Midwestern University, 19555 N 59th Ave, Glendale, AZ 85308, USA; ^4^The Marchand Institute for Minimally Invasive Surgery, 10238 E Hampton Ave, Suite 212, Mesa, AZ 85209, USA

## Abstract

Nonpuerperal uterine inversions are rare. Typically occurring in older women, they are most commonly due to transcervical mass expulsion. Diagnosis is often difficult because of vague symptomatology, presentation, and unknown course of the pathology. Surgical correction is often necessary in the presence of active bleeding or prolapse severity causing urinary retention. This case of nonpuerperal inversion presented to the emergency department with vaginal bleeding and mass protrusion. The examination was consistent with POPQ stage IV prolapse and uterine inversion secondary to cervical expulsion of multiple uterine fibroids. Because of full cervical dilation and concerns of ureteral injury with an extirpative procedure, vaginal myomectomy was performed with concomitant robotic uterosacral ligament hysteropexy. The operative procedure and postoperative course were uncomplicated, and discharge occurred on post-op day 1. She remained asymptomatic at the 6-month follow-up encounter. Though uterine preservation has been performed in cases of uterine inversion to maintain fertility, there are no reported cases of concomitant hysteropexy being completed for correction of POPQ stage IV prolapse simultaneously encountered. Additionally, the novel robotic approach has not been documented. This case illustrates the short-term success of robotic uterosacral hysteropexy as an additional option of care with potentially less morbidity when compared to hysterectomy for advanced stage uterine prolapse with nonpuerperal uterine inversion.

## 1. Introduction

While puerperal uterine inversions are an uncommon gynecologic event, nonpuerperal uterine inversions are extremely rare, reported 229 times between 1947 and 2017 [1]. Nonpuerperal inversions are caused by benign corpus uterine tumors 57.2% of the time, typically leiomyomas as demonstrated in this case. Their etiology is often multifactorial, due to rapid growth of the tumor, fundal location, thinning of the fundal wall, cervical dilation, and subsequent uterine expulsion [1]. Manual traction and increased intra-abdominal pressure can also contribute [2]. Women most often present with vaginal discharge, irregular uterine bleeding, pelvic discomfort, and anemia [3]. Depending on the acuity of the inversion, manual repositioning alone is possible. However, in chronic inversions, and those involving tumor growth, surgical intervention is often necessary [4]. Considering the average age of women who experience nonpuerperal inversion is above 45 years old, typically women have completed child bearing [5]. In 86.5% of patients, hysterectomy is performed for uterine inversion and is the most common corrective operative technique. This case presentation illustrates the success of surgical correction with a potentially less morbid procedure when POPQ stage IV prolapse is simultaneously encountered with nonpuerperal inversion [2].

## 2. Case Presentation

A 66-year-old without significant past medical or surgical history except pelvic organ prolapse presented to the emergency department complaining of vaginal bleeding and a mass protruding outside of the vaginal introitus. She had managed her chronic prolapse condition for the prior 5 years with self-reduction of the prolapse. The prolapse was not affecting her quality of life and she had not sought medical treatment prior to this emergency department encounter. An acute increase in vaginal bulge and associated vaginal bleeding precipitated the emergency encounter.

Upon presentation, an actively bleeding 15 cm mass was noted to be protruding from the vaginal introitus. Vital signs were stable, and the admission Hgb was 8 mg/dl. She was noted to be in urinary retention. With further evaluation and indwelling catheter placement, a decision was made to transfuse 2 units of packed red blood cells.

**Figure 1:**
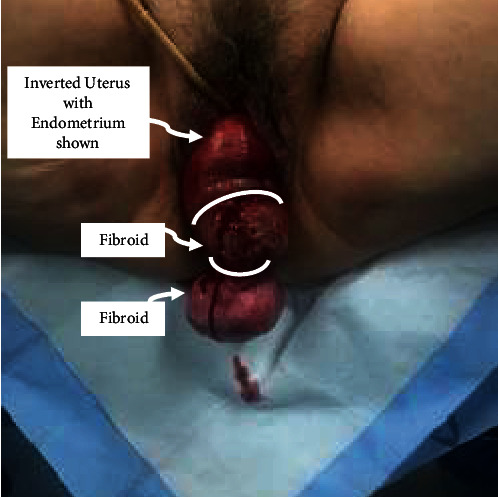
Preoperative uterine masses with complete uterovaginal prolapse and uterine inversion.

Closer examination of the vaginal mass revealed complete uterovaginal prolapse with uterine inversion (Figure 1). Two distinct masses consistent with uterine myomas were noted and found to have broad-based attachment to the inverted endometrial cavity. Active bleeding was encountered, and the patient was counseled and consented for surgical management. Because of the complete cervical dilation and advanced stage of prolapse, it was felt that total hysterectomy would be associated with greater risk of morbidity due to ureteral injury; therefore, a decision was made for uterine preservation. The patient expressed desire for native tissue correction of the prolapse.

Typical preoperative work was completed, and after adequate general endotracheal anesthesia was obtained, an examination under anesthesia confirmed the preoperative diagnosis. Starting vaginally, endometrial sampling was performed, and Ligassure® was utilized to obtain hemostasis and divide the broad-based masses from their insertion site. The prolapse was then manually reduced. A uterine manipulator was inserted and utilized to elevate the uterus to normal anatomic position.

Abdominal trocars were placed in a typical fashion, and the uterosacral ligaments were identified under tension. The peritoneum over both ligaments was opened and bilateral ureters were identified laterally. A monofilament polypropylene suture was passed from lateral to medial at the level of the ischial spines bilaterally. Each suture was then passed from lateral to medial of the posterior surface of the cervix with inclusion of the uterosacral ligament insertion site. Both sutures were then secured with care to avoid air knots.

**Figure 2:**
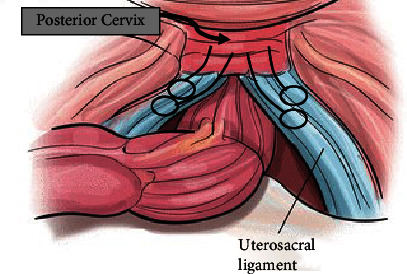
Uterosacral ligament hysteropexy [6].

A second suture of polydioxanone (delayed absorbable) was placed in a similar fashion 1 cm distal to the original suture placement bilaterally (Figure 2). These sutures were secured with close observation for ureteral compromise. Good apical support was obtained with point C -6. Cystourethroscopy was performed, and bilateral ureteral efflux was confirmed.

Overnight observation was performed with discharge the following morning after meeting post-op criteria. Minimal bleeding was noted. The Foley catheter was kept in place overnight and was discontinued the next day after a successful voiding trial. The patient was evaluated 2 and 6 weeks postoperatively. No immediate complications were noted, and she was asymptomatic. Pathology confirmed 2 benign leiomyomas and endometrial curettage. Six-month pelvic examination demonstrated continued apical support with point C at -6.

## 3. Discussion

Uterine inversions are typically categorized as obstetric, puerperal, or gynecologic nonpuerperal. Due to the rarity and nonspecific symptomatology of nonpuerperal inversions, diagnosis is often delayed. Patients frequently present with bleeding, discharge, pain, or symptomatic anemia. However, acute inversion patients may present with hypovolemic shock [3]. Physical exam often demonstrates a dark red, friable mass at the perineum, and differential diagnosis includes cervical, vaginal, or vulvar cancer. When presenting complaints and physical exam are not sufficient for diagnosis, imaging with MRI or ultrasound is indicated. Sagittal views will demonstrate a U-shaped uterine cavity with thickened fundus and axial views often demonstrate a “Bulls-eye” pattern [2]. This case did not require the additional imaging, and the acute nature of presentation necessitated prompt intervention.

An inversion staging system has been described in the literature, classifying this case as stage 4 inversion (Table 1) [1]. Systematic evaluation of an inversion should include biopsy of any mass if present with appropriate oncology referral if necessary. Patient age, fertility desires, desired route of surgical correction, manual reduction success, and morbidity of surgical options should all be considered and discussed with the patient.

Though hysterectomy is the most common surgical procedure performed for inversion, four surgical options for repositioning of the inverted fundus are described in the literature. Abdominal correction of the inverted fundus may be accomplished by a Haultain or Huntington technique, and vaginal correction of the inversion may be accomplished by a Kustner or Spinelli vaginal approach [3, 6].

This case allowed for a minimally invasive technique of correction, and, in our experience, is the first documented case of a nonpuerperal inversion with advanced stage uterine prolapse managed laparoscopically with robotic assistance. This surgical technique may be considered in management algorithms of nonpuerperal uterine eversion with advanced stage prolapse. Uterine preservation with hysteropexy may provide a less morbid surgical option when compared to hysterectomy.

## Figures and Tables

**Table 1:** Stages of genital inversion [1].

Stage	Description
1	Incomplete inversion, fundus is within the uterine cavity
2	Complete inversion, fundus is protruding through the cervix
3	Total inversion, fundus is protruding through the vulva
4	Total inversion, fundus is protruding through the vulva with vagina involved

## Data Availability

The data used for this case presentation is available in medical records at Star Clinic.

## References

[1] Rosa Silva B., de Oliveira Meller F., Uggioni M. L. (2018). Non-puerperal uterine inversion: a systematic review. *Gynecologic and Obstetric Investigation*.

[2] Rocconi R., Huh W. K., Chiang S. (2003). Postmenopausal uterine inversion associated with endometrial polyps. *Obstetrics and Gynecology*.

[3] Herath R. P., Patabendige M., Rashid M., Wijesinghe P. S. (2020). Nonpuerperal uterine inversion: what the gynaecologists need to know?. *Obstetrics and Gynecology International*.

[4] Kumari A., Vidhyarthi A., Salini K. M. (2016). Chronic uterine inversion secondary to submucous fibroid: a rare case report. *International Journal of Scientific Study*.

[5] Kouamé A., Koffi S. V., Adjoby R. (2015). Non-puerperal uterine inversion in a young woman: a case report. *Journal of the West African College of Surgeons*.

[6] Handa V. L., Van Le L. (2019). *Te Linde’s Operative Gynecology*.

